# Clinical validation of prospective liquid biopsy monitoring in patients with wild-type RAS metastatic colorectal cancer treated with FOLFIRI-cetuximab

**DOI:** 10.18632/oncotarget.13311

**Published:** 2016-11-11

**Authors:** Rodrigo A. Toledo, Antonio Cubillo, Estela Vega, Elena Garralda, Rafael Alvarez, Lisardo U. de la Varga, Jesús R. Pascual, Gema Sánchez, Francesca Sarno, Susana H. Prieto, Sofía Perea, Pedro P. Lopéz-Casas, Fernando López-Ríos, Manuel Hidalgo

**Affiliations:** ^1^ Centro Integral Oncológico Clara Campal (CIOCC), Madrid, Spain; ^2^ Universidad San Pablo CEU, Madrid, Spain; ^3^ Laboratorio de Dianas Terapeúticas, Madrid, Spain; ^4^ Spanish National Cancer Research Centre (CNIO), Madrid, Spain; ^5^ Beth Israel Deaconess Medical Center, Harvard Medical School, Boston, Massachusetts, USA

**Keywords:** colorectal cancer, cfDNA, liquid biopsy, anti-EGFR, cetuximab

## Abstract

Cancer genomics and translational medicine rely on the molecular profiling of patient's tumor obtained during surgery or biopsy. Alternatively, blood is a less invasive source of tumor DNA shed, amongst other ways, as cell-free DNA (cfDNA). Highly-sensitive assays capable to detect cancer genetic events from patient's blood plasma became popularly known as liquid biopsy (LqB). Importantly, retrospective studies including small number of selected patients with metastatic colorectal cancer (mCRC) patients treated with anti-EGFR therapy have shown LqB capable to detect the acquired clonal mutations in RAS genes leading to therapy resistance. However, the usefulness of LqB in the real-life clinical monitoring of these patients still lack additional validation on controlled studies. In this context, we designed a prospective LqB clinical trial to monitor newly diagnosed *KRAS* wild-type (wt) mCRC patients who received a standard FOLFIRI-cetuximab regimen. We used BEAMing technique for evaluate cfDNA mutations in *KRAS*, *NRAS, BRAF*, and *PIK3CA* in twenty-five patients during a 2-y period. A total of 2,178 cfDNA mutation analyses were performed and we observed that: a) continued wt circulating status was correlated with a prolonged response; b) smoldering increases in mutant cfDNA were correlated with acquired resistance; while c) mutation upsurge/explosion anticipated a remarkable clinical deterioration. The current study provides evidences, obtained for the first time in an unbiased and prospective manner, that reinforces the utility of LqB for monitoring mCRC patients.

## INTRODUCTION

Colorectal cancer (CRC) is the second most common neoplasm in humans, accounting for more than 1.3 million new cases and approximately 700,000 deaths per year [1]. Treatment with anti-EGFR antibodies prolongs overall survival in *RAS* wt mCRC patients. However, these patients ultimately progress, at least partially because of the emergence of mutations that occur in genes in the RAS pathway during treatment [2–5].

Liquid biopsy (LqB) is a blood exam that is capable of detecting circulating tumor cells (CTCs) and/or small fragments of cell-free tumor DNA (cfDNA), which are shed into the bloodstream from both primary and secondary neoplastic lesions. This new technology is considered a game-changing procedure because it represents a noninvasive alternative for identifying solid tumor heterogeneity. It also provides an assessment of cancer resistant sub-clones, and its results potentially reflect the molecular dynamics associated with tumor responsiveness and drug resistance [4–11]. Improvements in the technology have been made, and decreases are being observed in the turn-around time and costs of the procedure. It is therefore reasonable to anticipate that in the near future, hospitals and cancer centers will routinely offer LqB to cancer patients using in-house or commercially available kits and gene panels. At this time, however, it is essential to expand the information available regarding the usefulness and limitations of the LqB technique, especially the clinical interpretation of its results [6–9].

The aim of the present study was to gain additional knowledge regarding the clinical application of LqB by prospectively quantitating the temporal evolution of mutations in genes in the RAS pathway in cfDNA obtained from KRAS wt mCRC patients who were treated with first line FOLFIRI-cetuximab. We sought to relate the circulating genetic status of the patients with tumor genotypes, drug resistance, and predictions of clinical outcomes.

## RESULTS

The study and data collection were conducted between April 2013 and December 2015.

### Patient outcomes

**Figure 1:**
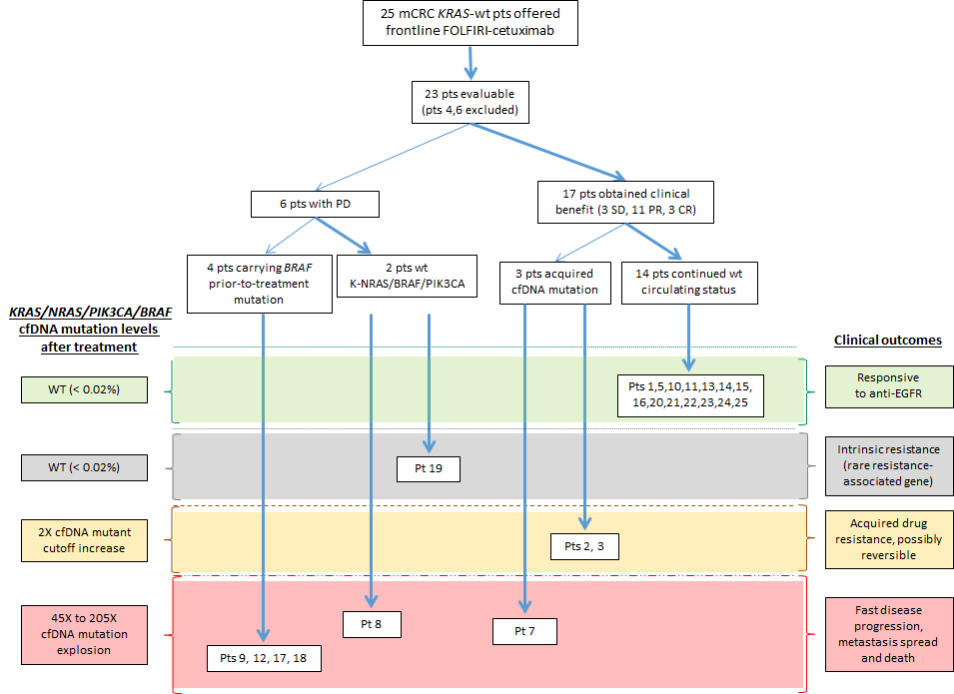
Flowchart of patient disposition A total of twenty-five patients were included in the study, and twenty-three patients were evaluable. Response to FOLFIRI-cetuximab and clinical outcome are shown to each patient, as well as tumor and cfDNA mutation status. Patient 8 had a driver mutation in an unknown resistance-associated gene and later had a *KRAS* cfDNA mutation explosion, therefore was classified accordingly.

Figure 1 displays the outcomes in the twenty-five wt *KRAS* mCRC patients who were included in the study. All cases were evaluated for tumor genotyping, and twenty-three cases were evaluated for drug responsiveness and clinical outcome. Patients 4 and 6 were excluded because insufficient plasma samples were collected after treatment.

Seventeen cases (17/23, 74%) showed a clinical benefit, including three patients with stable disease, eleven with a partial response, and three with a complete response. Of the patients who did not experience a clinical benefit, three carried *BRAF* mutations, and one carried *BRAF/PIK3CA* mutations in their tumor tissues prior to treatment. Importantly, these mutations were also detected in cfDNA baseline and on treatment plasma samples of these patients. When only the evaluated patients who had no *KRAS*/*NRAS*/*BRAF*/*PIK3CA* mutations prior to treatment were included, 89.5% of the patients (17/19) benefited from the treatment.

The remaining two patients presented continuous disease progression but had no tumor or circulating mutations in the analyzed genes prior to treatment. In these patients, disease progression was likely the result of a mutation in a rarer or unknown resistance-associated gene.

Detailed information regarding the clinical features, drug responsiveness, cfDNA prospective monitoring and interpretation of liquid biopsy results in the twenty-three evaluated patients is shown in Table 1.

**Table 1:** Clinical and genetic information of the twenty-three evaluable mCRC patients included in the study. A summary of the liquid biopsy results of each patient and its interpretation are provided

Patient	Primary tumor	Secondary lesions	Liquid biopsy and clinical results	Best response	Status at end of study	Interpretation
1	Rectal	Ganglionar	Continued wt cfDNA in all liquid biopsy analysis	SD	Alive with disease	Continued wt cfDNA status associated with prolonged response to FOLFIRI-cetuximab
2	Rectal	Liver	Initially wt but later cfDNA became mutated coinciding with liver metastasis grow. Patient was operated and went back to wt. Months later cfDNA became mutated again before tumor relapse could be observed by imaging exam. Another surgery was performed	PR	Alive without disease	Moderated levels of cfDNA mutation associated with acquired resistance. After-surgery increased levels associated with tumor relapse.
3	Colon	Ganglionar and lung	Initially wt but later became mutated when changed to cetuximab monotherapy and lung metastasis started grow. Re-use of FOLFIRI-cetuximab stop tumor grow and returned cfDNA results to wt	PR	Alive with disease	Moderated increase of cfDNA mutation associated with acquired resistance. Rechallenge with FOLFIRI-cetuximab zeroed cfDNA mutation levels and disease stabilized.
5	Rectal	Pelvis	Continued wt cfDNA in all liquid biopsy analysis	SD	Alive with disease	Continued wt cfDNA status associated with prolonged response to FOLFIRI-cetuximab
7	Rectal	Liver	Continued wt cfDNA until pre-surgery liver embolization. cfDNA *KRAS* mutation explosion associated with rapid metastasis spread and dead	PR	Deceased	Explosion of cfDNA *KRAS* mutation levels associated with rapid clinical deterioration.
8	Rectal	Liver	Continuous progression of the disease despite continued wt cfDNA in liquid biopsy analysis. cfDNA *KRAS* mutation explosion after cetuximab-afatinib treatment	PD	Deceased	Explosion of cfDNA *KRAS* mutation levels associated with rapid clinical deterioration.
9	Colon	Lung and peritoneal	Somatic *BRAF* mutation identified in high levels in cfDNA and substantially increased after FOLFIRI-cetuximab. Fast disease progression.	PD	Deceased	Explosion of cfDNA *BRAF* mutation levels associated with rapid clinical deterioration.
10	Rectal	Lung	Continued wt cfDNA in all liquid biopsy analysis	CR	Alive without disease	Continued wt cfDNA status associated with prolonged response to FOLFIRI-cetuximab
11	Rectal	Ganglionar	Continued wt cfDNA in all liquid biopsy analysis	CR	Alive without disease	Continued wt cfDNA status associated with prolonged response to FOLFIRI-cetuximab
12	Colon	Liver and ganglionar	Somatic *BRAF* mutation identified in high levels in cfDNA and substantially increased after FOLFIRI-cetuximab. Fast disease progression.	PD	Deceased	Explosion of cfDNA *BRAF* mutation levels associated with rapid clinical deterioration.
13	Colon	Liver	Continued wt cfDNA in all liquid biopsy analysis	PR	Alive with disease	Continued wt cfDNA status associated with prolonged response to FOLFIRI-cetuximab
14	Colon	Liver	Continued wt cfDNA in all liquid biopsy analysis	PR	Alive with disease	Continued wt cfDNA status associated with prolonged response to FOLFIRI-cetuximab
15	Colon	Ganglionar and lung	Continued wt cfDNA in all liquid biopsy analysis	PR	Alive without disease	Continued wt cfDNA status associated with prolonged response to FOLFIRI-cetuximab
16	Rectal	Liver	Continued wt cfDNA in all liquid biopsy analysis	PR	Alive with disease	Continued wt cfDNA status associated with prolonged response to FOLFIRI-cetuximab
17	Colon	Lung	Somatic *BRAF* mutation identified in high levels in cfDNA and substantially increased after FOLFIRI-cetuximab. Fast disease progression.	PD	Deceased	Explosion of cfDNA *BRAF* mutation levels associated with rapid clinical deterioration.
18	Colon	Ganglionar and liver	Somatic *BRAF* and *PIK3CA* mutations identified in high levels in cfDNA and substantially increased after FOLFIRI-cetuximab. Fast disease progression.	PD	Deceased	Explosion of cfDNA *BRAF* mutation levels associated with rapid clinical deterioration.
19	Colon	Skin	Continuous progression of the disease despite continued wt cfDNA in all liquid biopsy analysis	PD	Alive without disease	Mutation in a rarer anti-EGFR resistant-associated gene causing resistance and disease progression.
20	Colon	Peritoneal	Continued wt cfDNA in all liquid biopsy analysis	PR	Lost follow-up	Continued wt cfDNA status associated with prolonged response to FOLFIRI-cetuximab
21	Colon	Ganglionar	Continued wt cfDNA in all liquid biopsy analysis	PR	Alive without disease	Continued wt cfDNA status associated with prolonged response to FOLFIRI-cetuximab
22	Colon	Ganglionar	Continued wt cfDNA in all liquid biopsy analysis	PR	Alive with disease	Continued wt cfDNA status associated with prolonged response to FOLFIRI-cetuximab
23	Rectal	Ovary /peritoneal	Continued wt cfDNA in all liquid biopsy analysis	SD	Alive without disease	Continued wt cfDNA status associated with prolonged response to FOLFIRI-cetuximab
24	Colon	Ganglionar	Continued wt cfDNA in all liquid biopsy analysis	CR	Alive with disease	Continued wt cfDNA status associated with prolonged response to FOLFIRI-cetuximab
25	Colon	Liver	Continued wt cfDNA in all liquid biopsy analysis	PR	Alive without disease	Continued wt cfDNA status associated with prolonged response to FOLFIRI-cetuximab

### Sensitivity and specificity of BEAMing technique

**Figure 2:**
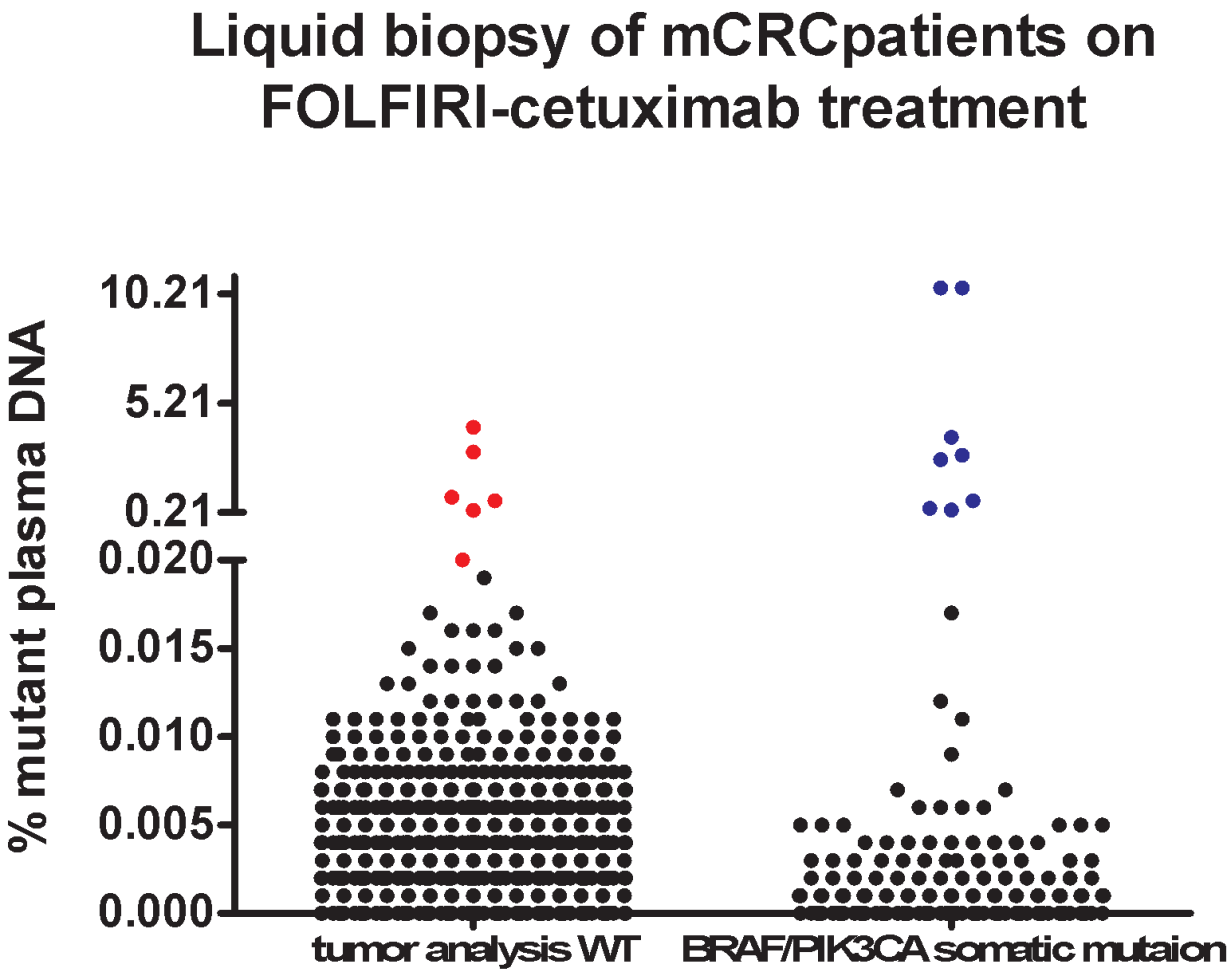
cfDNA mutation analyses of the twenty-five mCRC patients included in the study is shown divided in prior (**upper figure**) and after FOLFIRI-cetuximab treatment (**below figure**). Patients are separated accordingly to somatic mutation status (wt tumors shown in left panels, and BRAF/PIK3CA-mutated tumors in right panels) prior treatment. Stablished cfDNA mutation cutoff is 0,02%. Black dots are cfDNA mutation levels < 0,02%, blue dots correspond to prior treatment tumor mutations observed in the cfDNA at > 0,02% levels. Red dots are cfDNA mutation levels > 0,02% newly acquired during the FOLFIRI-cetuximab therapy.

We observed a perfect match (100% specificity and sensitivity of the BEAMing technique compared to Ion Torrent and Cobas FDA-approved tumor mutation assessment kits) in the results of our analysis of *KRAS*, *NRAS*, *PIK3CA* and *BRAF* mutations in tumor and prior treatment plasma samples. In accordance to tumor genotyping, the *BRAF* V600E mutation was found in the basal cfDNA of patient 9 (0.07% of plasma DNA fragments), patient 12 (10.47%), patient 17 (0.40%) and patient 18 (3.64%), and the *PIK3CA* H1047R mutation was found in the cfDNA of patient 18 (2.62%) (Figure 2A). The remaining 545 BEAMing mutation analyses that were performed using plasma samples prior to treatment were wt (Table S1).

The mean cfDNA value for the corresponding *BRAF*/*PIK3CA* alleles that were mutated prior to treatment was 3.44% ± 1.88 (N = 5), indicating a 172-fold higher rate than the 0.02% mutation cutoff value. When all twenty-two mutations were analyzed in the same four patients who a mutation prior to treatment, the mean values in the mutant cfDNA continued to be 10-fold higher than the mutation cutoff (0.20% ± 0.13; N = 88). However, the rate of cfDNA mutations in the patients without any prior *KRAS*/*NRAS*/*BRAF*/*PIK3CA* tumor mutations was 10-fold lower than the cutoff (0.002% ± 0.00014; N = 374).

Prior to treatment, the results of the cfDNA mutation analyses were statistically different between patients with *KRAS*/*NRAS*/*BRAF*/*PIK3CA* wt tumors and patients with tumors carrying *BRAF*/*PIK3CA* mutations (*p* < 0.0001 when only *BRAF*/*PIK3CA* mutated alleles were included in the analysis, and *p* = 0.0019 when all twenty-two alleles were included in the analysis; Figure 2A).

### cfDNA mutation levels and patient clinical outcomes

**Figure 3:**
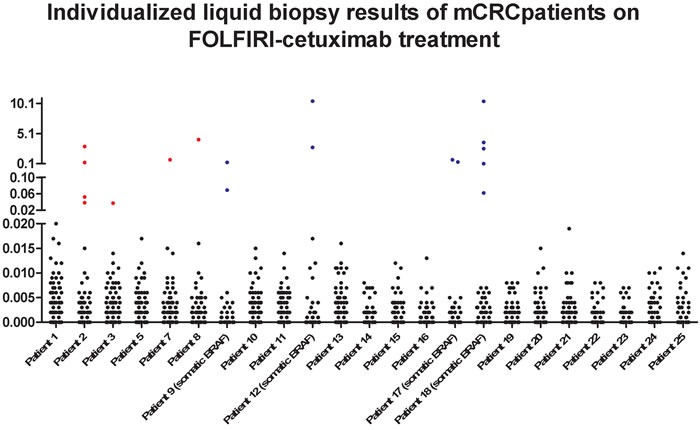
Results of cfDNA from the twenty-three evaluable mCRC patients of the study are shown Stablished cfDNA mutation cutoff is 0,02%. Black dots are cfDNA mutation levels < 0,02%, blue dots correspond to prior to treatment tumor mutations observed in the cfDNA at > 0,02% levels. Red dots are cfDNA mutation levels > 0,02% newly acquired during the FOLFIRI-cetuximab therapy. Patients 9, 12, 17 and 18 carried somatic *BRAF* mutation prior treatment.

We next investigated how changes in cfDNA mutation levels that occurred during FOLFIRI-cetuximab treatment (Figure 2B) were correlated with patients’ clinical outcomes. The individualized values of the plasma DNA mutations identified during the 2,178 BEAMing mutation analyses performed for this study are shown in Figure 3. In addition to the four patients with BRAF/PIK3CA mutations prior to treatment, the KRAS and PIK3CA cfDNAs became mutated in three and one additional patients, respectively. cfDNA status was correlated with clinical outcomes in three groups of patients: prolonged responders, patients with acquired resistance, and patients with progressive disease.

### Prolonged response to frontline anti-EGFR therapy and maintenance of wt cfDNA status

The median rate at which we identified DNA plasma “mutant” fragments in the seventeen patients who were good responders during treatment was 0.001%. Small oscillations were observed in these levels (standard deviation ± 0.002%). However, these results do not implicate changes in clinical or treatment responses. For example, plasma samples were obtained from Patient 1, at twelve different time points (resulting in a total of 264 mutation analyses), and the results showed that there were oscillations, but no cfDNA mutation value was higher than the 0.02% cutoff (Figure S1).

Of note, there were no statistically significant differences in the cfDNA profiles between the good responders who underwent surgery and those who continued with anti-EGFR therapy throughout the study period (*p* = 0.21).

### Acquired resistance to anti-EGFR treatment

**Figure 4:**
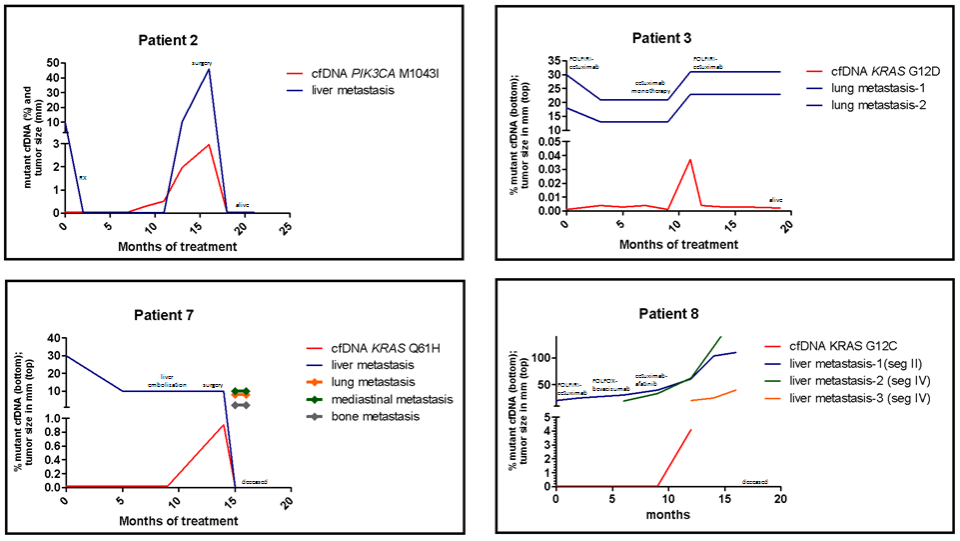
Treatment response and cfDNA status of four patients that acquired *KRAS/PIK3CA* during FOLFIRI-cetuximab treatment mutations are shown Patients 2 presented gradual increase of cfDNA *PIK3CA* mutation that anteceded disease relapse. Patient 3 had an intermediated increase in cfDNA *KRAS* mutation coinciding with tumor grow. Patients 7 and 8 suffered very rapid cfDNA mutation increase (mutation explosion) followed by fast metastasis spread and clinical deterioration that culminated in death. Clinical and genetic detailed information of each patient are described in the results section..

Changes in plasma DNA mutant fragments that were above the 0.02% cutoff reflected a clinical alteration that likely resulted from a switch in molecular dynamics. For example, Patient 2 responded to FOLFIRI-cetuximab, became suitable for radio surgery, maintained a disease-free state for 10 months and then relapses. A *PIK3CA* M1043I cfDNA level of 0.038% (2-fold the upper mutant cutoff limit) was observed in the plasma sample that was collected three months before the positive identification of disease relapse in an imaging exam. The patient underwent surgery, and at her last clinical follow-up, which occurred 31 months after she was initially included in the study, indicated that there were no signs of a new disease relapse (Figure 4, first panel)).

Patient 3 achieved a 50% reduction in the size of two lung metastases during the patient's initial ten months on FOLFIRI-cetuximab treatment. Because of its toxicity, the chemotherapy was then withdrawn. In the following two months, the patient was treated with anti-EGFR monotherapy, and an increase was observed in *KRAS* G12D cfDNA alleles from 0.001% to 0.037% (also 2-fold the mutant cutoff limit, as was observed in Patient 2). The reintroduction of the FOLFIRI-cetuximab treatment resulted in a decrease in *KRAS* G12D cfDNA levels to 0.002%-0.004% in the following months, and the disease was again considered to be controlled (Figure 4, second panel). The patient continued to maintain a controlled disease states at 30 months after she entered the study.

### An abrupt increase in *KRAS* cfDNA levels precedes clinical deterioration

Patient 7 achieved a partial response during cycle 4 of FOLFIRI-cetuximab treatment, and surgical intervention was then recommended. Pre-operative embolization of the left portal vein was performed followed by a right hepatectomy. CT scans performed one month after surgery revealed an absence of the disease, and treatment was considered successful. Two months later, the patient surprisingly presented with severe clinical deterioration, and follow-up scans revealed multiple pleuropulmonary, bone, and hepatic metastases as well as mediastinal lymph nodes that led to fatal respiratory failure. Similar cases of tumor flare-up after pre-hepatectomy embolization to treat mCRC have been reported [12, 13]. Importantly, patient 7's cfDNA *KRAS* Q61H levels abruptly increased from 0%-0.002% in plasma samples that were collected at baseline and after 6 months of treatment with FOLFIRI-cetuximab to 0.909% in plasma samples collected at two months after the hepatic embolization and a week before the hepatectomy (Figure 4, third panel).

A massive upsurge in *KRAS* G12C circulating mutations was also observed in Patient 8, and this upsurge was similarly correlated with rapid metastatic growth and clinical deterioration. After completing a standard treatment regimen, this patient was included in a clinical trial for a combination of cetuximab and afatinib. Subsequent plasma analyses revealed a rapid increase in the proportion of *KRAS* G12C cfDNA alleles from 0% to 4.42% and a rapid intensification of metastatic growth after the EGFR pathway was dually and strongly blocked. The disease progress culminated in the patient's death soon afterwards (Figure 4, fourth panel).

After observing that rapid upsurges in circulating mutant alleles were associated with poor clinical outcomes in wt mCRC patients, we sought to investigate whether the same pattern would be observed mCRC patients with mutations prior to treatment. These patients are known to be poorer responders to anti-EGFR therapy. For these analyses, we evaluated the basal and on-treatment cfDNA status of the four patients in our cohort who carried a prior to treatment tumor mutation in *BRAF* or *BRAF*/*PIK3CA*. As expected, these patients presented a quick clinical progression. After a brief decrease in plasma DNA mutation levels after treatment start, a remarkable upsurge in cfDNA mutation levels was observed in these patients during treatment (an average increase of 376%). For example, prior to treatment, patient 18 had cfDNA *BRAF* V600E and *PIK3CA* H1047R levels of 3.64% and 2.62%, respectively. These levels decreased to 0.11% and 0.06%, at three months after the onset of treatment but then increased to 16.07% and 10.47%, at six months after the onset of treatment, and this increase coincided with clinical deterioration. In addition, Patient 9 experienced an increase in *BRAF* V600E cfDNA levels of 488% within one month of the initiation of treatment.

**Figure 5:**
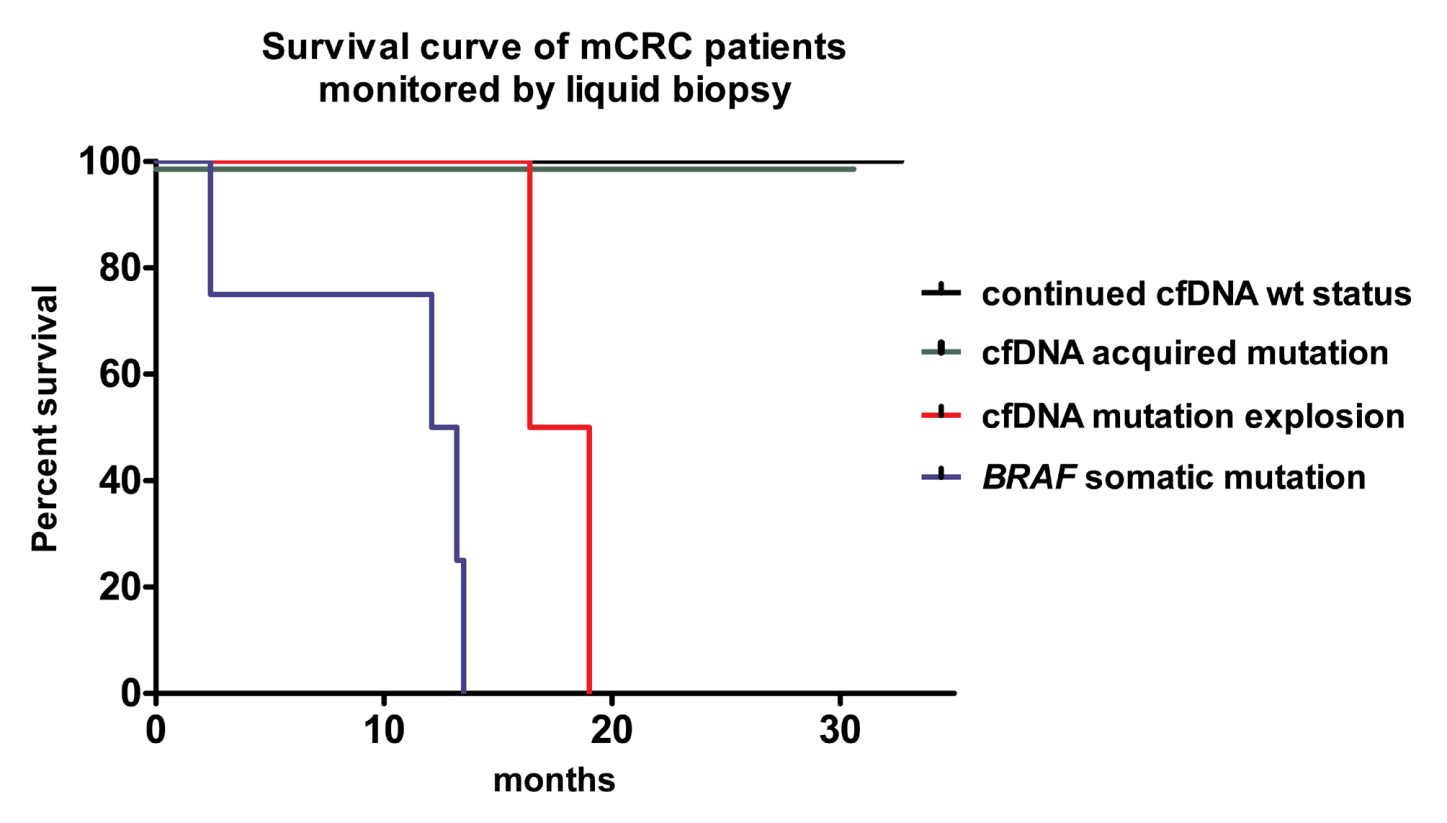
Kaplan-Meir survival curve of patients separated by somatic and cfDNA mutation status All six patients with *BRAF* somatic mutation or cfDNA mutation explosions progressed fast and died. None of the seventeen patients with continued wt cfDNA status (N = 15) or with intermediate/gradual increase in cfDNA mutations (N = 2) died during the study.

A Kaplan-Meir analysis showed that the survival curves in mCRC patients were significantly different when the patients were stratified according to genetic status. Patients carrying *BRAF* mutations prior to treatment (these mutations were also identified in samples collected at the basal timepoint and during treatment) and patients who experienced a cfDNA mutation explosion events progressed significantly faster than either patients with continued wt cfDNA status or patients who showed a gradual increase in cfDNA mutation levels that was reduced (zeroed) after retreatment with FOLFIRI-cetuximab or surgery (Log-rank test *p* < 0.0001, Figure 5).

## DISCUSSION

Liquid biopsy monitoring of cancer patients is a technically available and affordable procedure. However, our current knowledge regarding the interpretation of analyses of plasma mutations must be expanded before it can be routinely implemented into daily clinical use. The present study summarizes the results of a prospective study of liquid biopsies performed on *KRAS* wt mCRC patients who were treated with FOLFIRI-cetuximab as a frontline treatment.

Our results support concordance between the results of mutation analyses performed using cfDNA and those that use tumor tissue biopsies (the current standard approach used at most institutions). In accordance with previous large studies that have used BEAMing to genotype CRC patients [14], the results of the present study showed that the BEAMing technique provided very high sensibility and sensitivity when used for tumor genotyping (Table S1). The plasma samples obtained from included patients who carried a mutation in *BRAF*/*PIK3CA* prior to treatment contained 10- to 172-fold higher cfDNA mutation values than the 0.02% established mutation cutoff. On contrary, the levels of cfDNA mutations in patients without a prior *KRAS*/*NRAS*/*BRAF*/*PIK3CA* tumor mutation were 10-fold lower than the mutation cutoff (*p* = 0.002). These results confirm that liquid biopsy is a valuable non-invasive method for tumor mutation genotyping.

Originally, we sought to use cfDNA mutation levels as a real-time tool for prospectively monitoring the emergence of drug resistance in our cohort. Our results showed that the patients who initially responded to anti-EGFR therapy but later acquired resistance presented intermediate and gradually increasing levels of circulating mutant alleles. For example, the proportion of circulating mutant alleles in Patient 3 rose to 2X the mutant cutoff, and this increase coincided with the progression of the patient's lung metastatic lesions. Interestingly, maintaining anti-EGFR therapy while re-introducing chemotherapy stopped tumor growth and stabilized the disease. One possible interpretation for the response observed in this case is that the molecular dynamics of the tumor began to change, and this caused a moderate increase in the level of circulating mutant alleles. However, the tumor had not yet become irreversibly resistant. This finding is corroborated by a recent study by Siravegna *et al* [15] that suggested that continuing or re-challenging a patient with anti-EGFR therapy can be beneficial in some cases. It is not known, however, whether treatment with chemotherapy alone would achieve the same outcome. The role of maintaining the suppression of EGFR in the context of rising levels of cfDNA mutant alleles should be addressed in future clinical trials.

Another important point this study aimed to address was related to the possibility of using liquid biopsy results to predict long-term responses and good clinical outcomes. This assumption could not be examined in previous liquid biopsy studies because they included 2^nd^ to 4^th^ line mCRC patients who after started receiving anti-EGFR therapy rapidly became resistant (usually between 4-6 months after the onset of treatment)^4^. The present study included newly diagnosed, untreated, *KRAS* wt, advanced CRC patients who responded to FOLFORI-cetuximab for much longer periods and who therefore allowed us to gather, for the first time, valuable data related to the use of liquid biopsy in mCRC patients who displayed prolonged responses to first-line anti-EGFR therapy. Interestingly, the results showed that patients with long-term responses maintained a wt circulating status throughout the period during which they underwent anti-EGFR therapy. While these findings await validation in larger cohorts, our results strongly indicate that a wt cfDNA status is a potential biomarker of a prolonged good response in mCRC patients during treatment with anti-EGFR therapies.

In addition to the finding that wt cfDNA levels are potential biomarker for a continued response to FOLFIRI-cetuximab, it has also been observed that the opposite situation is also true: highly and rapidly increased levels of cfDNA mutations are a potential biomarker for a poor prognosis because these increases were followed by imminent clinical deterioration and the spread of metastases. For example, Patients 7 and 8 showed upsurges in circulating *KRAS* mutations from 0.001% to 0.909% and from 0% to 4%, respectively, over a short period of time, and these increases coincided with the clinical deterioration of the patient in both cases. Remarkably, while we observed an increase in mutations that was 2-fold the cutoff level in the plasma of the patients that developed gradually and reversible resistance, patients 7 and 8 displayed a very rapid 45- to 205-fold increase over the mutation cutoff level in their cfDNA levels. These upsurges were very intense and were likely associated with a change in tumor biology, and we therefore called them cfDNA “mutation explosions”. Importantly, the association of mutation explosions and clinical deterioration was also observed in the four mCRC patients who harbored *BRAF*/*PIK3CA* mutations prior to treatment. In these patients, the mutation upsurge averaged 376% after treatment using FOLFIRI-cetuximab and coincided with rapid disease progression..

To the best of our knowledge, the finding that this circulating mutation explosion “phenomenon” is a potential predictive biomarker of bad prognosis, has not been previously described. One possible explanation for the mutation explosion observed in Patient 7 is that a prior surgery-related embolization created a hypoxic environment that activated hypoxia-inducible transcription factors (HIFs), which themselves triggered metastatic cascades and molecular changes in the tumor that were much deeper in the tumor than those that occur in tumors with smoldering acquired resistance [16].

In light of this new finding, we strongly recommend that the observation of cfDNA mutation upsurges in a prospective follow up of a mCRC patient should raise a red flag. Selecting a liquid biopsy technology that has both high sensibility and sensitivity and a short turn-around time for results would enable a clinicians to promptly receive genetic results that would be potentially useful in prospective follow ups with the patient. Our results further indicate that it is also important to take costs into consideration because the number of liquid biopsy analyses that are required can be substantial during the follow-up period in patients receiving frontline anti-EGFR treatment. Another technical consideration is that assessing mutations in *KRAS/NRAS*/*BRAF*/*PIK3CA* was informative in all but two patients who progressed despite having a wt circulating status (2/22, 9%). The inclusion of genes that are more rarely involved in anti-EGFR resistance, such as *MET*, *ERBB2*, *FLT3*, *EGFR* and *MAP2K1* [19] in the panel of liquid biopsy genes could potentially identify mutations in these two “wt” patients. However, the cost-efficiency of including these markers should be further evaluated.

In summary, the results of the present study confirm that BEAMing is a high-efficiency method for tumor genotyping and for evaluating resistance to anti-EGFR treatment. Moreover, in this proof-of-concept prospective trial of liquid biopsy monitoring, we demonstrated for the first time that a continued wt circulating mutation status is a valuable biomarker of a prolonged tumor response to anti-EGFR therapy, while mutation explosion events predicted an eminent clinical deterioration in mCRC patients. Our findings should encourage the design of larger studies that focus on prospectively using cfDNA mutation analyses as a tool for monitoring drug responses and predicting clinical outcomes in newly diagnosed mCRC patients.

## PATIENTS AND METHODS

### Study design and patient eligibility

Adult patients who were newly diagnosed with untreated *KRAS* wt advanced CRC and who were candidates for first line chemotherapy were eligible. Patients received first line treatment with cetuximab at 400 mg/m^2^ on day 1 followed by 250 mg/m^2^ weekly thereafter and FOLFIRI (day 1: irinotecan, 180 mg/m^2^; folinic acid, 400 mg/m^2^; and fluorouracil, 400 mg/m^2^ *via* intravenous bolus; then 2,400 mg/m^2^ over 46 h *via* continuous infusion) every two weeks. Tumor responses were assessed every four cycles (2 months) using CT or MRI and classified according to RECIST criteria. Dose reductions and toxicity management were performed according to standard practices.

This study was approved by the institutional review board, conducted in accordance with the Declaration of Helsinki and the International Conference on Harmonization of Good Clinical Practice guidelines and registered inClinicalTrials.gov (NCT01943786). All patients gave prior written informed consent.

### Tumor sample mutation analysis

Samples from the primary tumor or metastatic lesions were collected by biopsy, and the following twenty-two hotspot mutations were analyzed using Ion Torrent™ Next-Generation Sequencing (Ion AmpliSeq™ Library Kit 2.0, Life Technologies, USA) or FDA-approved Cobas mutation kits (Roche Molecular Diagnosis, Swiss): *KRAS* (G12S/R/C/V/A/D, G13D, Q61H, and A146T), *NRAS* (Q61K/R/L/H), *BRAF* (V600E), and *PIK3CA* (E542K, E545K/G, Q546K, M1043I, and H1047Y/R/L).

### Liquid biopsy

Liquid biopsy was performed using the beads, emulsion, amplification and magnetics (BEAMing) technique as previously described [17, 18]. Plasma samples were collected immediately before treatment started and the periodically collected during each tumor evaluation. Frozen plasma samples were sent in dry-ice to Sysmex-Inostics in Germany, where the same *KRAS*/*NRAS*/*PIK3CA*/*BRAF* hot-spot mutations that were assessed in the tumor samples were genotyped in cfDNA using BEAMing. A detailed protocol for these procedures is included in the supplementary methods.

In accordance with the company´s experience, the sensitivity cutoff for mutation detection was set at a lower limit of 0.02% to avoid false positive results. Values below this level were considered wt results. As an internal quality control, a total of 509 mutation analyses were performed using samples that were previously genotyped by Sysmex, and a 100% match was obtained for all twenty-two mutations.

### Statistical analyses

*T*-tests were used to assess differences in cfDNA mutation levels between mCRC patients with *KRAS*/*NRAS*/*PIK3CA*/*BRAF* wt tumors prior to treatment and patients who had tumors carrying *BRAF* or *BRAF*/*PIK3CA* mutations before therapy began. A survival analysis was performed using Kaplan-Meier tests. The log-rank test was used to verify whether differences between survival curves were statistically different. GraphPad Prism software was used for all statistical analyses. A *p*-value lower than 0.05 was considered statistically significant.

## SUPPLEMENTARY MATERIALS FIGURES AND TABLES





## Abbreviations

LqB: liquid biopsy
WT: wild type
CRC: colorectal cancer
mCRC: metastatic CRC
cfDNA: plasma cell-free DNA
CTC: circulating tumor cells
FOLFIRI: Folinic acid, Fluorouracil, Irinotecan
EGFR: Epidermal growth factor receptor
Cetuximab: EGFR monoclonal antibody
HIF: hypoxia inducible factor
BEAMing: beads, emulsion, amplification and magnetics technique

## References

[1] Siegel R, Naishadham D, Jemal A (2012). Cancer statistics. CA: a cancer journal for clinicians.

[2] Van Cutsem E, Köhne CH, Hitre E, Zaluski J, Chang Chien CR, Makhson A, D'Haens G, Pintér T, Lim R, Bodoky G, Roh JK, Folprecht G, Ruff P (2009). Cetuximab and chemotherapy as initial treatment for metastatic colorectal cancer. New England Journal of Medicine.

[3] Karapetis CS, Khambata-Ford S, Jonker DJ, O'Callaghan CJ, Tu D, Tebbutt NC, Simes RJ, Chalchal H, Shapiro JD, Robitaille S, Price TJ, Shepherd L, Au HJ (2008). K-ras mutations and benefit from cetuximab in advanced colorectal cancer. New England Journal of Medicine.

[4] Douillard JY1, Oliner KS, Siena S, Tabernero J, Burkes R, Barugel M, Humblet Y, Bodoky G, Cunningham D, Jassem J, Rivera F, Kocákova I, Ruff P (2013). Panitumumab-FOLFOX4 treatment and RAS mutations in colorectal cancer. New England Journal of Medicine.

[5] Diaz LA, Williams RT, Wu J, Kinde I, Hecht JR, Berlin J, Allen B, Bozic I, Reiter JG, Nowak MA, Kinzler KW, Oliner KS, Vogelstein B (2012). The molecular evolution of acquired resistance to targeted EGFR blockade in colorectal cancers. Nature.

[6] Misale S, Yaeger R, Hobor S, Scala E, Janakiraman M, Liska D, Valtorta E, Schiavo R, Buscarino M, Siravegna G, Bencardino K, Cercek A, Chen CT (2012). Emergence of KRAS mutations and acquired resistance to anti-EGFR therapy in colorectal cancer. Nature.

[7] Crowley E, Di Nicolantonio F, Loupakis F, Bardelli A (2013). Liquid biopsy: monitoring cancer-genetics in the blood. Nature Reviews Clinical Oncology.

[8] Lee JY, Qing X, Xiumin W, Yali B, Chi S, Bak SH, Lee HY, Sun JM, Lee SH, Ahn JS, Cho EK, Kim DW, Kim HR Longitudinal monitoring of EGFR mutations in plasma predicts outcomes of NSCLC patients treated with EGFR TKIs: Korean Lung Cancer Consortium (KLCC-12-02). Oncotarget.

[9] Murtaza M, Dawson SJ, Tsui DW, Gale D, Forshew T, Piskorz AM, Parkinson C, Chin SF, Kingsbury Z, Wong AS, Marass F, Humphray S, Hadfield J (2013). Non-invasive analysis of acquired resistance to cancer therapy by sequencing of plasma DNA. Nature.

[10] Bettegowda C, Sausen M, Leary RJ, Kinde I, Wang Y, Agrawal N, Bartlett BR, Wang H, Luber B, Alani RM, Antonarakis ES, Azad NS, Bardelli A (2014). Detection of circulating tumor DNA in early-and late-stage human malignancies. Science translational medicine.

[11] Dienstmann R, Salazar R, Tabernero J (2014). Overcoming Resistance to Anti-EGFR Therapy in Colorectal Cancer. In American Society of Clinical Oncology educational book/ASCO American Society of Clinical Oncology Meeting.

[12] de Graaf W, van den Esschert JW, van Lienden KP, van Gulik TM (2009). Induction of tumor growth after preoperative portal vein embolization: is it a real problem?. Annals of surgical oncology.

[13] Hoekstra LT, van Lienden KP, Doets A, Busch OR, Gouma DJ, van Gulik TM (2012). Tumor progression after preoperative portal vein embolization. Annals of surgery.

[14] Tabernero J, Lenz HJ, Siena S, Sobrero A, Falcone A, Ychou M, Humblet Y, Bouché O, Mineur L, Barone C, Adenis A, Yoshino T, Goldberg RM (2015). Analysis of circulating DNA and protein biomarkers to predict the clinical activity of regorafenib and assess prognosis in patients with metastatic colorectal cancer: a retrospective, exploratory analysis of the CORRECT trial. The Lancet Oncology.

[15] Siravegna G, Mussolin B, Buscarino M, Corti G, Cassingena A, Crisafulli G, Ponzetti A, Cremolini C, Amatu A, Lauricella C, Lamba S, Hobor S, Avallone A (2015). Clonal evolution and resistance to EGFR blockade in the blood of colorectal cancer patients. Nature medicine.

[16] Harris AL (2002). Hypoxia—a key regulatory factor in tumour growth. Nature Reviews Cancer.

[17] Li M, Diehl F, Dressman D, Vogelstein B, Kinzler KW (2006). BEAMing up for detection and quantification of rare sequence variants. Nature Methods.

[18] F1 Diehl, Schmidt K, Choti MA, Romans K, Goodman S, Li M, Thornton K, Agrawal N, Sokoll L, Szabo SA, Kinzler KW, Vogelstein B, Diaz LA (2008). Circulating mutant DNA to assess tumor dynamics. Nature medicine.

[19] Medico E, Russo M, Picco G, Cancelliere C, Valtorta E, Corti G, Buscarino M, Isella C, Lamba S, Martinoglio B, Veronese S, Siena S, Sartore-Bianchi A (2015). The molecular landscape of colorectal cancer cell lines unveils clinically actionable kinase targets. Nature communications.

