# Supplementary material for: Clinical validation of prospective liquid biopsy monitoring in patients with wild-type RAS metastatic colorectal cancer treated with FOLFIRI-cetuximab
Source: Oncotarget. 2016 Nov 11;8(21):35289–300. doi: 10.18632/oncotarget.13311 (PMC5471055; doi:10.18632/oncotarget.13311)
Supplement: Supplementary file 1 [file oncotarget-08-35289-s001.pdf]

Clinical validation of prospective liquid biopsy monitoring in patients with wild-type RAS metastatic colorectal cancer treated with FOLFIRI-cetuximab

Supplementary Material

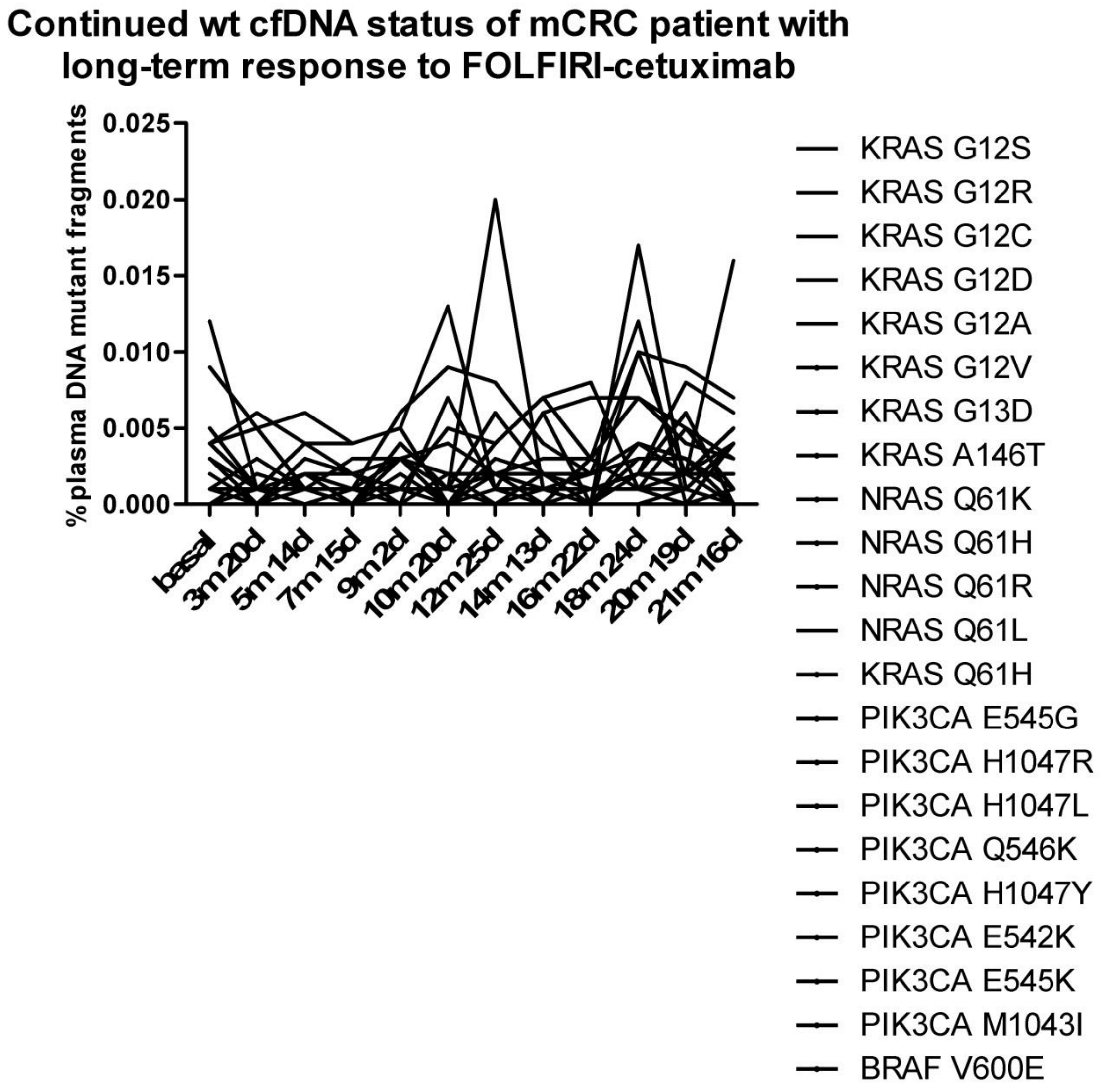

Figure S1

Continued wt status (<0,02%) in a total of 264 cfDNA mutation levels in a long-term responder to FOLFIRI-cetuximab treatment.
