# Supplementary material for: Clinical validation of prospective liquid biopsy monitoring in patients with wild-type RAS metastatic colorectal cancer treated with FOLFIRI-cetuximab
Source: Oncotarget. 2016 Nov 11;8(21):35289–300. doi: 10.18632/oncotarget.13311 (PMC5471055; doi:10.18632/oncotarget.13311)
Supplement: Supplementary file 2 [file oncotarget-08-35289-s002.docx]

**Table S1**

Results of mutation analyzes performed in tumor and plasma samples prior FOLFIRI-cetuximab treatment.

| **#** | **Sample ID** | **Gene** | **Mutation analyzed** | **Tumor** | **Plasma** | **Concordance** |
| --- | --- | --- | --- | --- | --- | --- |
| 1 | Patient 01 | KRAS | A146T | wild type | wild type | Y |
| 2 | Patient 01 | PIK3CA | E542K | wild type | wild type | Y |
| 3 | Patient 01 | PIK3CA | E545G | wild type | wild type | Y |
| 4 | Patient 01 | PIK3CA | E545K | wild type | wild type | Y |
| 5 | Patient 01 | KRAS | G12A | wild type | wild type | Y |
| 6 | Patient 01 | KRAS | G12C | wild type | wild type | Y |
| 7 | Patient 01 | KRAS | G12D | wild type | wild type | Y |
| 8 | Patient 01 | KRAS | G12R | wild type | wild type | Y |
| 9 | Patient 01 | KRAS | G12S | wild type | wild type | Y |
| 10 | Patient 01 | KRAS | G12V | wild type | wild type | Y |
| 11 | Patient 01 | KRAS | G13D | wild type | wild type | Y |
| 12 | Patient 01 | PIK3CA | H1047L | wild type | wild type | Y |
| 13 | Patient 01 | PIK3CA | H1047R | wild type | wild type | Y |
| 14 | Patient 01 | PIK3CA | H1047Y | wild type | wild type | Y |
| 15 | Patient 01 | PIK3CA | M1043I | wild type | wild type | Y |
| 16 | Patient 01 | PIK3CA | Q546K | wild type | wild type | Y |
| 17 | Patient 01 | KRAS | Q61H | wild type | wild type | Y |
| 18 | Patient 01 | NRAS | Q61H | ND | wild type | NA |
| 19 | Patient 01 | NRAS | Q61K | ND | wild type | NA |
| 20 | Patient 01 | NRAS | Q61L | ND | wild type | NA |
| 21 | Patient 01 | NRAS | Q61R | ND | wild type | NA |
| 22 | Patient 01 | BRAF | V600E | wild type | wild type | Y |
| 23 | Patient 02 | KRAS | A146T | wild type | wild type | Y |
| 24 | Patient 02 | PIK3CA | E542K | ND | wild type | NA |
| 25 | Patient 02 | PIK3CA | E545G | ND | wild type | NA |
| 26 | Patient 02 | PIK3CA | E545K | ND | wild type | NA |
| 27 | Patient 02 | KRAS | G12A | wild type | wild type | Y |
| 28 | Patient 02 | KRAS | G12C | wild type | wild type | Y |
| 29 | Patient 02 | KRAS | G12D | wild type | wild type | Y |
| 30 | Patient 02 | KRAS | G12R | wild type | wild type | Y |
| 31 | Patient 02 | KRAS | G12S | wild type | wild type | Y |
| 32 | Patient 02 | KRAS | G12V | wild type | wild type | Y |
| 33 | Patient 02 | KRAS | G13D | wild type | wild type | Y |
| 34 | Patient 02 | PIK3CA | H1047L | ND | wild type | NA |
| 35 | Patient 02 | PIK3CA | H1047R | ND | wild type | NA |
| 36 | Patient 02 | PIK3CA | H1047Y | ND | wild type | NA |
| 37 | Patient 02 | PIK3CA | M1043I | ND | wild type | NA |
| 38 | Patient 02 | PIK3CA | Q546K | ND | wild type | NA |
| 39 | Patient 02 | KRAS | Q61H | wild type | wild type | Y |
| 40 | Patient 02 | NRAS | Q61H | ND | wild type | NA |
| 41 | Patient 02 | NRAS | Q61K | ND | wild type | NA |
| 42 | Patient 02 | NRAS | Q61L | ND | wild type | NA |
| 43 | Patient 02 | NRAS | Q61R | ND | wild type | NA |
| 44 | Patient 02 | BRAF | V600E | wild type | wild type | Y |
| 45 | Patient 03 | KRAS | A146T | wild type | wild type | Y |
| 46 | Patient 03 | PIK3CA | E542K | wild type | wild type | Y |
| 47 | Patient 03 | PIK3CA | E545G | wild type | wild type | Y |
| 48 | Patient 03 | PIK3CA | E545K | wild type | wild type | Y |
| 49 | Patient 03 | KRAS | G12A | wild type | wild type | Y |
| 50 | Patient 03 | KRAS | G12C | wild type | wild type | Y |
| 51 | Patient 03 | KRAS | G12D | wild type | wild type | Y |
| 52 | Patient 03 | KRAS | G12R | wild type | wild type | Y |
| 53 | Patient 03 | KRAS | G12S | wild type | wild type | Y |
| 54 | Patient 03 | KRAS | G12V | wild type | wild type | Y |
| 55 | Patient 03 | KRAS | G13D | wild type | wild type | Y |
| 56 | Patient 03 | PIK3CA | H1047L | wild type | wild type | Y |
| 57 | Patient 03 | PIK3CA | H1047R | wild type | wild type | Y |
| 58 | Patient 03 | PIK3CA | H1047Y | wild type | wild type | Y |
| 59 | Patient 03 | PIK3CA | M1043I | wild type | wild type | Y |
| 60 | Patient 03 | PIK3CA | Q546K | wild type | wild type | Y |
| 61 | Patient 03 | KRAS | Q61H | wild type | wild type | Y |
| 62 | Patient 03 | NRAS | Q61H | wild type | wild type | Y |
| 63 | Patient 03 | NRAS | Q61K | wild type | wild type | Y |
| 64 | Patient 03 | NRAS | Q61L | wild type | wild type | Y |
| 65 | Patient 03 | NRAS | Q61R | wild type | wild type | Y |
| 66 | Patient 03 | BRAF | V600E | wild type | wild type | Y |
| 67 | Patient 04 | KRAS | A146T | wild type | wild type | Y |
| 68 | Patient 04 | PIK3CA | E542K | wild type | wild type | Y |
| 69 | Patient 04 | PIK3CA | E545G | wild type | wild type | Y |
| 70 | Patient 04 | PIK3CA | E545K | wild type | wild type | Y |
| 71 | Patient 04 | KRAS | G12A | wild type | wild type | Y |
| 72 | Patient 04 | KRAS | G12C | wild type | wild type | Y |
| 73 | Patient 04 | KRAS | G12D | wild type | wild type | Y |
| 74 | Patient 04 | KRAS | G12R | wild type | wild type | Y |
| 75 | Patient 04 | KRAS | G12S | wild type | wild type | Y |
| 76 | Patient 04 | KRAS | G12V | wild type | wild type | Y |
| 77 | Patient 04 | KRAS | G13D | wild type | wild type | Y |
| 78 | Patient 04 | PIK3CA | H1047L | wild type | wild type | Y |
| 79 | Patient 04 | PIK3CA | H1047R | wild type | wild type | Y |
| 80 | Patient 04 | PIK3CA | H1047Y | wild type | wild type | Y |
| 81 | Patient 04 | PIK3CA | M1043I | wild type | wild type | Y |
| 82 | Patient 04 | PIK3CA | Q546K | wild type | wild type | Y |
| 83 | Patient 04 | KRAS | Q61H | wild type | wild type | Y |
| 84 | Patient 04 | NRAS | Q61H | ND | wild type | NA |
| 85 | Patient 04 | NRAS | Q61K | ND | wild type | NA |
| 86 | Patient 04 | NRAS | Q61L | ND | wild type | NA |
| 87 | Patient 04 | NRAS | Q61R | ND | wild type | NA |
| 88 | Patient 04 | BRAF | V600E | wild type | wild type | Y |
| 89 | Patient 05 | KRAS | A146T | wild type | wild type | Y |
| 90 | Patient 05 | PIK3CA | E542K | ND | wild type | NA |
| 91 | Patient 05 | PIK3CA | E545G | ND | wild type | NA |
| 92 | Patient 05 | PIK3CA | E545K | ND | wild type | NA |
| 93 | Patient 05 | KRAS | G12A | wild type | wild type | Y |
| 94 | Patient 05 | KRAS | G12C | wild type | wild type | Y |
| 95 | Patient 05 | KRAS | G12D | wild type | wild type | Y |
| 96 | Patient 05 | KRAS | G12R | wild type | wild type | Y |
| 97 | Patient 05 | KRAS | G12S | wild type | wild type | Y |
| 98 | Patient 05 | KRAS | G12V | wild type | wild type | Y |
| 99 | Patient 05 | KRAS | G13D | wild type | wild type | Y |
| 100 | Patient 05 | PIK3CA | H1047L | ND | wild type | NA |
| 101 | Patient 05 | PIK3CA | H1047R | ND | wild type | NA |
| 102 | Patient 05 | PIK3CA | H1047Y | ND | wild type | NA |
| 103 | Patient 05 | PIK3CA | M1043I | ND | wild type | NA |
| 104 | Patient 05 | PIK3CA | Q546K | ND | wild type | NA |
| 105 | Patient 05 | KRAS | Q61H | wild type | wild type | Y |
| 106 | Patient 05 | NRAS | Q61H | ND | wild type | NA |
| 107 | Patient 05 | NRAS | Q61K | ND | wild type | NA |
| 108 | Patient 05 | NRAS | Q61L | ND | wild type | NA |
| 109 | Patient 05 | NRAS | Q61R | ND | wild type | NA |
| 110 | Patient 05 | BRAF | V600E | ND | wild type | NA |
| 111 | Patient 06 | KRAS | A146T | wild type | wild type | Y |
| 112 | Patient 06 | PIK3CA | E542K | ND | wild type | NA |
| 113 | Patient 06 | PIK3CA | E545G | ND | wild type | NA |
| 114 | Patient 06 | PIK3CA | E545K | ND | wild type | NA |
| 115 | Patient 06 | KRAS | G12A | wild type | wild type | Y |
| 116 | Patient 06 | KRAS | G12C | wild type | wild type | Y |
| 117 | Patient 06 | KRAS | G12D | wild type | wild type | Y |
| 118 | Patient 06 | KRAS | G12R | wild type | wild type | Y |
| 119 | Patient 06 | KRAS | G12S | wild type | wild type | Y |
| 120 | Patient 06 | KRAS | G12V | wild type | wild type | Y |
| 121 | Patient 06 | KRAS | G13D | wild type | wild type | Y |
| 122 | Patient 06 | PIK3CA | H1047L | ND | wild type | NA |
| 123 | Patient 06 | PIK3CA | H1047R | ND | wild type | NA |
| 124 | Patient 06 | PIK3CA | H1047Y | ND | wild type | NA |
| 125 | Patient 06 | PIK3CA | M1043I | ND | wild type | NA |
| 126 | Patient 06 | PIK3CA | Q546K | ND | wild type | NA |
| 127 | Patient 06 | KRAS | Q61H | wild type | wild type | Y |
| 128 | Patient 06 | NRAS | Q61H | ND | wild type | NA |
| 129 | Patient 06 | NRAS | Q61K | ND | wild type | NA |
| 130 | Patient 06 | NRAS | Q61L | ND | wild type | NA |
| 131 | Patient 06 | NRAS | Q61R | ND | wild type | NA |
| 132 | Patient 06 | BRAF | V600E | ND | wild type | NA |
| 133 | Patient 07 | KRAS | A146T | wild type | wild type | Y |
| 134 | Patient 07 | PIK3CA | E542K | ND | wild type | NA |
| 135 | Patient 07 | PIK3CA | E545G | ND | wild type | NA |
| 136 | Patient 07 | PIK3CA | E545K | ND | wild type | NA |
| 137 | Patient 07 | KRAS | G12A | wild type | wild type | Y |
| 138 | Patient 07 | KRAS | G12C | wild type | wild type | Y |
| 139 | Patient 07 | KRAS | G12D | wild type | wild type | Y |
| 140 | Patient 07 | KRAS | G12R | wild type | wild type | Y |
| 141 | Patient 07 | KRAS | G12S | wild type | wild type | Y |
| 142 | Patient 07 | KRAS | G12V | wild type | wild type | Y |
| 143 | Patient 07 | KRAS | G13D | wild type | wild type | Y |
| 144 | Patient 07 | PIK3CA | H1047L | ND | wild type | NA |
| 145 | Patient 07 | PIK3CA | H1047R | ND | wild type | NA |
| 146 | Patient 07 | PIK3CA | H1047Y | ND | wild type | NA |
| 147 | Patient 07 | PIK3CA | M1043I | ND | wild type | NA |
| 148 | Patient 07 | PIK3CA | Q546K | ND | wild type | NA |
| 149 | Patient 07 | KRAS | Q61H | wild type | wild type | Y |
| 150 | Patient 07 | NRAS | Q61H | ND | wild type | NA |
| 151 | Patient 07 | NRAS | Q61K | ND | wild type | NA |
| 152 | Patient 07 | NRAS | Q61L | ND | mutated | NA |
| 153 | Patient 07 | NRAS | Q61R | ND | wild type | NA |
| 154 | Patient 07 | BRAF | V600E | wild type | wild type | Y |
| 155 | Patient 08 | KRAS | A146T | wild type | wild type | Y |
| 156 | Patient 08 | PIK3CA | E542K | wild type | wild type | Y |
| 157 | Patient 08 | PIK3CA | E545G | wild type | wild type | Y |
| 158 | Patient 08 | PIK3CA | E545K | wild type | wild type | Y |
| 159 | Patient 08 | KRAS | G12A | wild type | wild type | Y |
| 160 | Patient 08 | KRAS | G12C | wild type | wild type | Y |
| 161 | Patient 08 | KRAS | G12D | wild type | wild type | Y |
| 162 | Patient 08 | KRAS | G12R | wild type | wild type | Y |
| 163 | Patient 08 | KRAS | G12S | wild type | wild type | Y |
| 164 | Patient 08 | KRAS | G12V | wild type | wild type | Y |
| 165 | Patient 08 | KRAS | G13D | wild type | wild type | Y |
| 166 | Patient 08 | PIK3CA | H1047L | wild type | wild type | Y |
| 167 | Patient 08 | PIK3CA | H1047R | wild type | wild type | Y |
| 168 | Patient 08 | PIK3CA | H1047Y | wild type | wild type | Y |
| 169 | Patient 08 | PIK3CA | M1043I | wild type | wild type | Y |
| 170 | Patient 08 | PIK3CA | Q546K | wild type | wild type | Y |
| 171 | Patient 08 | KRAS | Q61H | wild type | wild type | Y |
| 172 | Patient 08 | NRAS | Q61H | ND | wild type | NA |
| 173 | Patient 08 | NRAS | Q61K | ND | wild type | NA |
| 174 | Patient 08 | NRAS | Q61L | ND | wild type | NA |
| 175 | Patient 08 | NRAS | Q61R | ND | wild type | NA |
| 176 | Patient 08 | BRAF | V600E | wild type | wild type | Y |
| 177 | Patient 09 | KRAS | A146T | wild type | wild type | Y |
| 178 | Patient 09 | PIK3CA | E542K | wild type | wild type | Y |
| 179 | Patient 09 | PIK3CA | E545G | wild type | wild type | Y |
| 180 | Patient 09 | PIK3CA | E545K | wild type | wild type | Y |
| 181 | Patient 09 | KRAS | G12A | wild type | wild type | Y |
| 182 | Patient 09 | KRAS | G12C | wild type | wild type | Y |
| 183 | Patient 09 | KRAS | G12D | wild type | wild type | Y |
| 184 | Patient 09 | KRAS | G12R | wild type | wild type | Y |
| 185 | Patient 09 | KRAS | G12S | wild type | wild type | Y |
| 186 | Patient 09 | KRAS | G12V | wild type | wild type | Y |
| 187 | Patient 09 | KRAS | G13D | wild type | wild type | Y |
| 188 | Patient 09 | PIK3CA | H1047L | wild type | wild type | Y |
| 189 | Patient 09 | PIK3CA | H1047R | wild type | wild type | Y |
| 190 | Patient 09 | PIK3CA | H1047Y | wild type | wild type | Y |
| 191 | Patient 09 | PIK3CA | M1043I | wild type | wild type | Y |
| 192 | Patient 09 | PIK3CA | Q546K | wild type | wild type | Y |
| 193 | Patient 09 | KRAS | Q61H | wild type | wild type | Y |
| 194 | Patient 09 | NRAS | Q61H | wild type | wild type | Y |
| 195 | Patient 09 | NRAS | Q61K | wild type | wild type | Y |
| 196 | Patient 09 | NRAS | Q61L | wild type | wild type | Y |
| 197 | Patient 09 | NRAS | Q61R | wild type | wild type | Y |
| 198 | Patient 09 | BRAF | V600E | mutated | mutated | Y |
| 199 | Patient 10 | KRAS | A146T | wild type | wild type | Y |
| 200 | Patient 10 | PIK3CA | E542K | wild type | wild type | Y |
| 201 | Patient 10 | PIK3CA | E545G | wild type | wild type | Y |
| 202 | Patient 10 | PIK3CA | E545K | wild type | wild type | Y |
| 203 | Patient 10 | KRAS | G12A | wild type | wild type | Y |
| 204 | Patient 10 | KRAS | G12C | wild type | wild type | Y |
| 205 | Patient 10 | KRAS | G12D | wild type | wild type | Y |
| 206 | Patient 10 | KRAS | G12R | wild type | wild type | Y |
| 207 | Patient 10 | KRAS | G12S | wild type | wild type | Y |
| 208 | Patient 10 | KRAS | G12V | wild type | wild type | Y |
| 209 | Patient 10 | KRAS | G13D | wild type | wild type | Y |
| 210 | Patient 10 | PIK3CA | H1047L | wild type | wild type | Y |
| 211 | Patient 10 | PIK3CA | H1047R | wild type | wild type | Y |
| 212 | Patient 10 | PIK3CA | H1047Y | wild type | wild type | Y |
| 213 | Patient 10 | PIK3CA | M1043I | wild type | wild type | Y |
| 214 | Patient 10 | PIK3CA | Q546K | wild type | wild type | Y |
| 215 | Patient 10 | KRAS | Q61H | wild type | wild type | Y |
| 216 | Patient 10 | NRAS | Q61H | ND | wild type | NA |
| 217 | Patient 10 | NRAS | Q61K | ND | wild type | NA |
| 218 | Patient 10 | NRAS | Q61L | ND | wild type | NA |
| 219 | Patient 10 | NRAS | Q61R | ND | wild type | NA |
| 220 | Patient 10 | BRAF | V600E | wild type | wild type | Y |
| 221 | Patient 11 | KRAS | A146T | wild type | wild type | Y |
| 222 | Patient 11 | PIK3CA | E542K | wild type | wild type | Y |
| 223 | Patient 11 | PIK3CA | E545G | wild type | wild type | Y |
| 224 | Patient 11 | PIK3CA | E545K | wild type | wild type | Y |
| 225 | Patient 11 | KRAS | G12A | wild type | wild type | Y |
| 226 | Patient 11 | KRAS | G12C | wild type | wild type | Y |
| 227 | Patient 11 | KRAS | G12D | wild type | wild type | Y |
| 228 | Patient 11 | KRAS | G12R | wild type | wild type | Y |
| 229 | Patient 11 | KRAS | G12S | wild type | wild type | Y |
| 230 | Patient 11 | KRAS | G12V | wild type | wild type | Y |
| 231 | Patient 11 | KRAS | G13D | wild type | wild type | Y |
| 232 | Patient 11 | PIK3CA | H1047L | wild type | wild type | Y |
| 233 | Patient 11 | PIK3CA | H1047R | wild type | wild type | Y |
| 234 | Patient 11 | PIK3CA | H1047Y | wild type | wild type | Y |
| 235 | Patient 11 | PIK3CA | M1043I | wild type | wild type | Y |
| 236 | Patient 11 | PIK3CA | Q546K | wild type | wild type | Y |
| 237 | Patient 11 | KRAS | Q61H | wild type | wild type | Y |
| 238 | Patient 11 | NRAS | Q61H | wild type | wild type | Y |
| 239 | Patient 11 | NRAS | Q61K | wild type | wild type | Y |
| 240 | Patient 11 | NRAS | Q61L | wild type | wild type | Y |
| 241 | Patient 11 | NRAS | Q61R | wild type | wild type | Y |
| 242 | Patient 11 | BRAF | V600E | wild type | wild type | Y |
| 243 | Patient 12 | KRAS | A146T | wild type | wild type | Y |
| 244 | Patient 12 | PIK3CA | E542K | ND | wild type | NA |
| 245 | Patient 12 | PIK3CA | E545G | ND | wild type | NA |
| 246 | Patient 12 | PIK3CA | E545K | ND | wild type | NA |
| 247 | Patient 12 | KRAS | G12A | wild type | wild type | Y |
| 248 | Patient 12 | KRAS | G12C | wild type | wild type | Y |
| 249 | Patient 12 | KRAS | G12D | wild type | wild type | Y |
| 250 | Patient 12 | KRAS | G12R | wild type | wild type | Y |
| 251 | Patient 12 | KRAS | G12S | wild type | wild type | Y |
| 252 | Patient 12 | KRAS | G12V | wild type | wild type | Y |
| 253 | Patient 12 | KRAS | G13D | wild type | wild type | Y |
| 254 | Patient 12 | PIK3CA | H1047L | ND | wild type | NA |
| 255 | Patient 12 | PIK3CA | H1047R | ND | wild type | NA |
| 256 | Patient 12 | PIK3CA | H1047Y | ND | wild type | NA |
| 257 | Patient 12 | PIK3CA | M1043I | ND | wild type | NA |
| 258 | Patient 12 | PIK3CA | Q546K | ND | wild type | NA |
| 259 | Patient 12 | KRAS | Q61H | wild type | wild type | Y |
| 260 | Patient 12 | NRAS | Q61H | ND | wild type | NA |
| 261 | Patient 12 | NRAS | Q61K | ND | wild type | NA |
| 262 | Patient 12 | NRAS | Q61L | ND | wild type | NA |
| 263 | Patient 12 | NRAS | Q61R | ND | wild type | NA |
| 264 | Patient 12 | BRAF | V600E | mutated | mutated | Y |
| 265 | Patient 13 | KRAS | A146T | wild type | wild type | Y |
| 266 | Patient 13 | PIK3CA | E542K | wild type | wild type | Y |
| 267 | Patient 13 | PIK3CA | E545G | wild type | wild type | Y |
| 268 | Patient 13 | PIK3CA | E545K | wild type | wild type | Y |
| 269 | Patient 13 | KRAS | G12A | wild type | wild type | Y |
| 270 | Patient 13 | KRAS | G12C | wild type | wild type | Y |
| 271 | Patient 13 | KRAS | G12D | wild type | wild type | Y |
| 272 | Patient 13 | KRAS | G12R | wild type | wild type | Y |
| 273 | Patient 13 | KRAS | G12S | wild type | wild type | Y |
| 274 | Patient 13 | KRAS | G12V | wild type | wild type | Y |
| 275 | Patient 13 | KRAS | G13D | wild type | wild type | Y |
| 276 | Patient 13 | PIK3CA | H1047L | wild type | wild type | Y |
| 277 | Patient 13 | PIK3CA | H1047R | wild type | wild type | Y |
| 278 | Patient 13 | PIK3CA | H1047Y | wild type | wild type | Y |
| 279 | Patient 13 | PIK3CA | M1043I | wild type | wild type | Y |
| 280 | Patient 13 | PIK3CA | Q546K | wild type | wild type | Y |
| 281 | Patient 13 | KRAS | Q61H | wild type | wild type | Y |
| 282 | Patient 13 | NRAS | Q61H | ND | wild type | NA |
| 283 | Patient 13 | NRAS | Q61K | ND | wild type | NA |
| 284 | Patient 13 | NRAS | Q61L | ND | wild type | NA |
| 285 | Patient 13 | NRAS | Q61R | ND | wild type | NA |
| 286 | Patient 13 | BRAF | V600E | wild type | wild type | Y |
| 287 | Patient 14 | KRAS | A146T | wild type | wild type | Y |
| 288 | Patient 14 | PIK3CA | E542K | ND | wild type | NA |
| 289 | Patient 14 | PIK3CA | E545G | ND | wild type | NA |
| 290 | Patient 14 | PIK3CA | E545K | ND | wild type | NA |
| 291 | Patient 14 | KRAS | G12A | wild type | wild type | Y |
| 292 | Patient 14 | KRAS | G12C | wild type | wild type | Y |
| 293 | Patient 14 | KRAS | G12D | wild type | wild type | Y |
| 294 | Patient 14 | KRAS | G12R | wild type | wild type | Y |
| 295 | Patient 14 | KRAS | G12S | wild type | wild type | Y |
| 296 | Patient 14 | KRAS | G12V | wild type | wild type | Y |
| 297 | Patient 14 | KRAS | G13D | wild type | wild type | Y |
| 298 | Patient 14 | PIK3CA | H1047L | ND | wild type | NA |
| 299 | Patient 14 | PIK3CA | H1047R | ND | wild type | NA |
| 300 | Patient 14 | PIK3CA | H1047Y | ND | wild type | NA |
| 301 | Patient 14 | PIK3CA | M1043I | ND | wild type | NA |
| 302 | Patient 14 | PIK3CA | Q546K | ND | wild type | NA |
| 303 | Patient 14 | KRAS | Q61H | wild type | wild type | Y |
| 304 | Patient 14 | NRAS | Q61H | ND | wild type | NA |
| 305 | Patient 14 | NRAS | Q61K | ND | wild type | NA |
| 306 | Patient 14 | NRAS | Q61L | ND | wild type | NA |
| 307 | Patient 14 | NRAS | Q61R | ND | wild type | NA |
| 308 | Patient 14 | BRAF | V600E | wild type | wild type | Y |
| 309 | Patient 15 | KRAS | A146T | wild type | wild type | Y |
| 310 | Patient 15 | PIK3CA | E542K | wild type | wild type | Y |
| 311 | Patient 15 | PIK3CA | E545G | wild type | wild type | Y |
| 312 | Patient 15 | PIK3CA | E545K | wild type | wild type | Y |
| 313 | Patient 15 | KRAS | G12A | wild type | wild type | Y |
| 314 | Patient 15 | KRAS | G12C | wild type | wild type | Y |
| 315 | Patient 15 | KRAS | G12D | wild type | wild type | Y |
| 316 | Patient 15 | KRAS | G12R | wild type | wild type | Y |
| 317 | Patient 15 | KRAS | G12S | wild type | wild type | Y |
| 318 | Patient 15 | KRAS | G12V | wild type | wild type | Y |
| 319 | Patient 15 | KRAS | G13D | wild type | wild type | Y |
| 320 | Patient 15 | PIK3CA | H1047L | wild type | wild type | Y |
| 321 | Patient 15 | PIK3CA | H1047R | wild type | wild type | Y |
| 322 | Patient 15 | PIK3CA | H1047Y | wild type | wild type | Y |
| 323 | Patient 15 | PIK3CA | M1043I | wild type | wild type | Y |
| 324 | Patient 15 | PIK3CA | Q546K | wild type | wild type | Y |
| 325 | Patient 15 | KRAS | Q61H | wild type | wild type | Y |
| 326 | Patient 15 | NRAS | Q61H | wild type | wild type | Y |
| 327 | Patient 15 | NRAS | Q61K | wild type | wild type | Y |
| 328 | Patient 15 | NRAS | Q61L | wild type | wild type | Y |
| 329 | Patient 15 | NRAS | Q61R | wild type | wild type | Y |
| 330 | Patient 15 | BRAF | V600E | wild type | wild type | Y |
| 331 | Patient 16 | KRAS | A146T | wild type | wild type | Y |
| 332 | Patient 16 | PIK3CA | E542K | wild type | wild type | Y |
| 333 | Patient 16 | PIK3CA | E545G | wild type | wild type | Y |
| 334 | Patient 16 | PIK3CA | E545K | wild type | wild type | Y |
| 335 | Patient 16 | KRAS | G12A | wild type | wild type | Y |
| 336 | Patient 16 | KRAS | G12C | wild type | wild type | Y |
| 337 | Patient 16 | KRAS | G12D | wild type | wild type | Y |
| 338 | Patient 16 | KRAS | G12R | wild type | wild type | Y |
| 339 | Patient 16 | KRAS | G12S | wild type | wild type | Y |
| 340 | Patient 16 | KRAS | G12V | wild type | wild type | Y |
| 341 | Patient 16 | KRAS | G13D | wild type | wild type | Y |
| 342 | Patient 16 | PIK3CA | H1047L | wild type | wild type | Y |
| 343 | Patient 16 | PIK3CA | H1047R | wild type | wild type | Y |
| 344 | Patient 16 | PIK3CA | H1047Y | wild type | wild type | Y |
| 345 | Patient 16 | PIK3CA | M1043I | wild type | wild type | Y |
| 346 | Patient 16 | PIK3CA | Q546K | wild type | wild type | Y |
| 347 | Patient 16 | KRAS | Q61H | wild type | wild type | Y |
| 348 | Patient 16 | NRAS | Q61H | ND | wild type | NA |
| 349 | Patient 16 | NRAS | Q61K | ND | wild type | NA |
| 350 | Patient 16 | NRAS | Q61L | ND | wild type | NA |
| 351 | Patient 16 | NRAS | Q61R | ND | wild type | NA |
| 352 | Patient 16 | BRAF | V600E | wild type | wild type | Y |
| 353 | Patient 17 | KRAS | A146T | wild type | wild type | Y |
| 354 | Patient 17 | PIK3CA | E542K | ND | wild type | NA |
| 355 | Patient 17 | PIK3CA | E545G | ND | wild type | NA |
| 356 | Patient 17 | PIK3CA | E545K | ND | wild type | NA |
| 357 | Patient 17 | KRAS | G12A | wild type | wild type | Y |
| 358 | Patient 17 | KRAS | G12C | wild type | wild type | Y |
| 359 | Patient 17 | KRAS | G12D | wild type | wild type | Y |
| 360 | Patient 17 | KRAS | G12R | wild type | wild type | Y |
| 361 | Patient 17 | KRAS | G12S | wild type | wild type | Y |
| 362 | Patient 17 | KRAS | G12V | wild type | wild type | Y |
| 363 | Patient 17 | KRAS | G13D | wild type | wild type | Y |
| 364 | Patient 17 | PIK3CA | H1047L | ND | wild type | NA |
| 365 | Patient 17 | PIK3CA | H1047R | ND | wild type | NA |
| 366 | Patient 17 | PIK3CA | H1047Y | ND | wild type | NA |
| 367 | Patient 17 | PIK3CA | M1043I | ND | wild type | NA |
| 368 | Patient 17 | PIK3CA | Q546K | ND | wild type | NA |
| 369 | Patient 17 | KRAS | Q61H | wild type | wild type | Y |
| 370 | Patient 17 | NRAS | Q61H | wild type | wild type | Y |
| 371 | Patient 17 | NRAS | Q61K | wild type | wild type | Y |
| 372 | Patient 17 | NRAS | Q61L | wild type | wild type | Y |
| 373 | Patient 17 | NRAS | Q61R | wild type | wild type | Y |
| 374 | Patient 17 | BRAF | V600E | mutated | mutated | Y |
| 375 | Patient 18 | KRAS | A146T | wild type | wild type | Y |
| 376 | Patient 18 | PIK3CA | E542K | wild type | wild type | Y |
| 377 | Patient 18 | PIK3CA | E545G | wild type | wild type | Y |
| 378 | Patient 18 | PIK3CA | E545K | wild type | wild type | Y |
| 379 | Patient 18 | KRAS | G12A | wild type | wild type | Y |
| 380 | Patient 18 | KRAS | G12C | wild type | wild type | Y |
| 381 | Patient 18 | KRAS | G12D | wild type | wild type | Y |
| 382 | Patient 18 | KRAS | G12R | wild type | wild type | Y |
| 383 | Patient 18 | KRAS | G12S | wild type | wild type | Y |
| 384 | Patient 18 | KRAS | G12V | wild type | wild type | Y |
| 385 | Patient 18 | KRAS | G13D | wild type | wild type | Y |
| 386 | Patient 18 | PIK3CA | H1047L | wild type | wild type | Y |
| 387 | Patient 18 | PIK3CA | H1047R | mutated | mutated | Y |
| 388 | Patient 18 | PIK3CA | H1047Y | wild type | wild type | Y |
| 389 | Patient 18 | PIK3CA | M1043I | wild type | wild type | Y |
| 390 | Patient 18 | PIK3CA | Q546K | wild type | wild type | Y |
| 391 | Patient 18 | KRAS | Q61H | wild type | wild type | Y |
| 392 | Patient 18 | NRAS | Q61H | wild type | wild type | Y |
| 393 | Patient 18 | NRAS | Q61K | wild type | wild type | Y |
| 394 | Patient 18 | NRAS | Q61L | wild type | wild type | Y |
| 395 | Patient 18 | NRAS | Q61R | wild type | wild type | Y |
| 396 | Patient 18 | BRAF | V600E | mutated | mutated | Y |
| 397 | Patient 19 | KRAS | A146T | wild type | wild type | Y |
| 398 | Patient 19 | PIK3CA | E542K | wild type | wild type | Y |
| 399 | Patient 19 | PIK3CA | E545G | wild type | wild type | Y |
| 400 | Patient 19 | PIK3CA | E545K | wild type | wild type | Y |
| 401 | Patient 19 | KRAS | G12A | wild type | wild type | Y |
| 402 | Patient 19 | KRAS | G12C | wild type | wild type | Y |
| 403 | Patient 19 | KRAS | G12D | wild type | wild type | Y |
| 404 | Patient 19 | KRAS | G12R | wild type | wild type | Y |
| 405 | Patient 19 | KRAS | G12S | wild type | wild type | Y |
| 406 | Patient 19 | KRAS | G12V | wild type | wild type | Y |
| 407 | Patient 19 | KRAS | G13D | wild type | wild type | Y |
| 408 | Patient 19 | PIK3CA | H1047L | wild type | wild type | Y |
| 409 | Patient 19 | PIK3CA | H1047R | wild type | wild type | Y |
| 410 | Patient 19 | PIK3CA | H1047Y | wild type | wild type | Y |
| 411 | Patient 19 | PIK3CA | M1043I | wild type | wild type | Y |
| 412 | Patient 19 | PIK3CA | Q546K | wild type | wild type | Y |
| 413 | Patient 19 | KRAS | Q61H | wild type | wild type | Y |
| 414 | Patient 19 | NRAS | Q61H | wild type | wild type | Y |
| 415 | Patient 19 | NRAS | Q61K | wild type | wild type | Y |
| 416 | Patient 19 | NRAS | Q61L | wild type | wild type | Y |
| 417 | Patient 19 | NRAS | Q61R | wild type | wild type | Y |
| 418 | Patient 19 | BRAF | V600E | wild type | wild type | Y |
| 419 | Patient 20 | KRAS | A146T | wild type | wild type | Y |
| 420 | Patient 20 | PIK3CA | E542K | wild type | wild type | Y |
| 421 | Patient 20 | PIK3CA | E545G | wild type | wild type | Y |
| 422 | Patient 20 | PIK3CA | E545K | wild type | wild type | Y |
| 423 | Patient 20 | KRAS | G12A | wild type | wild type | Y |
| 424 | Patient 20 | KRAS | G12C | wild type | wild type | Y |
| 425 | Patient 20 | KRAS | G12D | wild type | wild type | Y |
| 426 | Patient 20 | KRAS | G12R | wild type | wild type | Y |
| 427 | Patient 20 | KRAS | G12S | wild type | wild type | Y |
| 428 | Patient 20 | KRAS | G12V | wild type | wild type | Y |
| 429 | Patient 20 | KRAS | G13D | wild type | wild type | Y |
| 430 | Patient 20 | PIK3CA | H1047L | wild type | wild type | Y |
| 431 | Patient 20 | PIK3CA | H1047R | wild type | wild type | Y |
| 432 | Patient 20 | PIK3CA | H1047Y | wild type | wild type | Y |
| 433 | Patient 20 | PIK3CA | M1043I | wild type | wild type | Y |
| 434 | Patient 20 | PIK3CA | Q546K | wild type | wild type | Y |
| 435 | Patient 20 | KRAS | Q61H | wild type | wild type | Y |
| 436 | Patient 20 | NRAS | Q61H | wild type | wild type | Y |
| 437 | Patient 20 | NRAS | Q61K | wild type | wild type | Y |
| 438 | Patient 20 | NRAS | Q61L | wild type | wild type | Y |
| 439 | Patient 20 | NRAS | Q61R | wild type | wild type | Y |
| 440 | Patient 20 | BRAF | V600E | wild type | wild type | Y |
| 441 | Patient 21 | KRAS | A146T | wild type | wild type | Y |
| 442 | Patient 21 | PIK3CA | E542K | wild type | wild type | Y |
| 443 | Patient 21 | PIK3CA | E545G | wild type | wild type | Y |
| 444 | Patient 21 | PIK3CA | E545K | wild type | wild type | Y |
| 445 | Patient 21 | KRAS | G12A | wild type | wild type | Y |
| 446 | Patient 21 | KRAS | G12C | wild type | wild type | Y |
| 447 | Patient 21 | KRAS | G12D | wild type | wild type | Y |
| 448 | Patient 21 | KRAS | G12R | wild type | wild type | Y |
| 449 | Patient 21 | KRAS | G12S | wild type | wild type | Y |
| 450 | Patient 21 | KRAS | G12V | wild type | wild type | Y |
| 451 | Patient 21 | KRAS | G13D | wild type | wild type | Y |
| 452 | Patient 21 | PIK3CA | H1047L | wild type | wild type | Y |
| 453 | Patient 21 | PIK3CA | H1047R | wild type | wild type | Y |
| 454 | Patient 21 | PIK3CA | H1047Y | wild type | wild type | Y |
| 455 | Patient 21 | PIK3CA | M1043I | wild type | wild type | Y |
| 456 | Patient 21 | PIK3CA | Q546K | wild type | wild type | Y |
| 457 | Patient 21 | KRAS | Q61H | wild type | wild type | Y |
| 458 | Patient 21 | NRAS | Q61H | wild type | wild type | Y |
| 459 | Patient 21 | NRAS | Q61K | wild type | wild type | Y |
| 460 | Patient 21 | NRAS | Q61L | wild type | wild type | Y |
| 461 | Patient 21 | NRAS | Q61R | wild type | wild type | Y |
| 462 | Patient 21 | BRAF | V600E | wild type | wild type | Y |
| 463 | Patient 22 | KRAS | A146T | wild type | wild type | Y |
| 464 | Patient 22 | PIK3CA | E542K | wild type | wild type | Y |
| 465 | Patient 22 | PIK3CA | E545G | wild type | wild type | Y |
| 466 | Patient 22 | PIK3CA | E545K | wild type | wild type | Y |
| 467 | Patient 22 | KRAS | G12A | wild type | wild type | Y |
| 468 | Patient 22 | KRAS | G12C | wild type | wild type | Y |
| 469 | Patient 22 | KRAS | G12D | wild type | wild type | Y |
| 470 | Patient 22 | KRAS | G12R | wild type | wild type | Y |
| 471 | Patient 22 | KRAS | G12S | wild type | wild type | Y |
| 472 | Patient 22 | KRAS | G12V | wild type | wild type | Y |
| 473 | Patient 22 | KRAS | G13D | wild type | wild type | Y |
| 474 | Patient 22 | PIK3CA | H1047L | wild type | wild type | Y |
| 475 | Patient 22 | PIK3CA | H1047R | wild type | wild type | Y |
| 476 | Patient 22 | PIK3CA | H1047Y | wild type | wild type | Y |
| 477 | Patient 22 | PIK3CA | M1043I | wild type | wild type | Y |
| 478 | Patient 22 | PIK3CA | Q546K | wild type | wild type | Y |
| 479 | Patient 22 | KRAS | Q61H | wild type | wild type | Y |
| 480 | Patient 22 | NRAS | Q61H | wild type | wild type | Y |
| 481 | Patient 22 | NRAS | Q61K | wild type | wild type | Y |
| 482 | Patient 22 | NRAS | Q61L | wild type | wild type | Y |
| 483 | Patient 22 | NRAS | Q61R | wild type | wild type | Y |
| 484 | Patient 22 | BRAF | V600E | wild type | wild type | Y |
| 485 | Patient 23 | KRAS | A146T | wild type | wild type | Y |
| 486 | Patient 23 | PIK3CA | E542K | ND | wild type | NA |
| 487 | Patient 23 | PIK3CA | E545G | ND | wild type | NA |
| 488 | Patient 23 | PIK3CA | E545K | ND | wild type | NA |
| 489 | Patient 23 | KRAS | G12A | wild type | wild type | Y |
| 490 | Patient 23 | KRAS | G12C | wild type | wild type | Y |
| 491 | Patient 23 | KRAS | G12D | wild type | wild type | Y |
| 492 | Patient 23 | KRAS | G12R | wild type | wild type | Y |
| 493 | Patient 23 | KRAS | G12S | wild type | wild type | Y |
| 494 | Patient 23 | KRAS | G12V | wild type | wild type | Y |
| 495 | Patient 23 | KRAS | G13D | wild type | wild type | Y |
| 496 | Patient 23 | PIK3CA | H1047L | ND | wild type | NA |
| 497 | Patient 23 | PIK3CA | H1047R | ND | wild type | NA |
| 498 | Patient 23 | PIK3CA | H1047Y | ND | wild type | NA |
| 499 | Patient 23 | PIK3CA | M1043I | ND | wild type | NA |
| 500 | Patient 23 | PIK3CA | Q546K | ND | wild type | NA |
| 501 | Patient 23 | KRAS | Q61H | wild type | wild type | Y |
| 502 | Patient 23 | NRAS | Q61H | wild type | wild type | Y |
| 503 | Patient 23 | NRAS | Q61K | wild type | wild type | Y |
| 504 | Patient 23 | NRAS | Q61L | wild type | wild type | Y |
| 505 | Patient 23 | NRAS | Q61R | wild type | wild type | Y |
| 506 | Patient 23 | BRAF | V600E | wild type | wild type | Y |
| 507 | Patient 24 | KRAS | A146T | wild type | wild type | Y |
| 508 | Patient 24 | PIK3CA | E542K | ND | wild type | NA |
| 509 | Patient 24 | PIK3CA | E545G | ND | wild type | NA |
| 510 | Patient 24 | PIK3CA | E545K | ND | wild type | NA |
| 511 | Patient 24 | KRAS | G12A | wild type | wild type | Y |
| 512 | Patient 24 | KRAS | G12C | wild type | wild type | Y |
| 513 | Patient 24 | KRAS | G12D | wild type | wild type | Y |
| 514 | Patient 24 | KRAS | G12R | wild type | wild type | Y |
| 515 | Patient 24 | KRAS | G12S | wild type | wild type | Y |
| 516 | Patient 24 | KRAS | G12V | wild type | wild type | Y |
| 517 | Patient 24 | KRAS | G13D | wild type | wild type | Y |
| 518 | Patient 24 | PIK3CA | H1047L | ND | wild type | NA |
| 519 | Patient 24 | PIK3CA | H1047R | ND | wild type | NA |
| 520 | Patient 24 | PIK3CA | H1047Y | ND | wild type | NA |
| 521 | Patient 24 | PIK3CA | M1043I | ND | wild type | NA |
| 522 | Patient 24 | PIK3CA | Q546K | ND | wild type | NA |
| 523 | Patient 24 | KRAS | Q61H | wild type | wild type | Y |
| 524 | Patient 24 | NRAS | Q61H | wild type | wild type | Y |
| 525 | Patient 24 | NRAS | Q61K | wild type | wild type | Y |
| 526 | Patient 24 | NRAS | Q61L | wild type | wild type | Y |
| 527 | Patient 24 | NRAS | Q61R | wild type | wild type | Y |
| 528 | Patient 24 | BRAF | V600E | wild type | wild type | Y |
| 529 | Patient 25 | KRAS | A146T | wild type | wild type | Y |
| 530 | Patient 25 | PIK3CA | E542K | ND | wild type | NA |
| 531 | Patient 25 | PIK3CA | E545G | ND | wild type | NA |
| 532 | Patient 25 | PIK3CA | E545K | ND | wild type | NA |
| 533 | Patient 25 | KRAS | G12A | wild type | wild type | Y |
| 534 | Patient 25 | KRAS | G12C | wild type | wild type | Y |
| 535 | Patient 25 | KRAS | G12D | wild type | wild type | Y |
| 536 | Patient 25 | KRAS | G12R | wild type | wild type | Y |
| 537 | Patient 25 | KRAS | G12S | wild type | wild type | Y |
| 538 | Patient 25 | KRAS | G12V | wild type | wild type | Y |
| 539 | Patient 25 | KRAS | G13D | wild type | wild type | Y |
| 540 | Patient 25 | PIK3CA | H1047L | ND | wild type | NA |
| 541 | Patient 25 | PIK3CA | H1047R | ND | wild type | NA |
| 542 | Patient 25 | PIK3CA | H1047Y | ND | wild type | NA |
| 543 | Patient 25 | PIK3CA | M1043I | ND | wild type | NA |
| 544 | Patient 25 | PIK3CA | Q546K | ND | wild type | NA |
| 545 | Patient 25 | KRAS | Q61H | wild type | wild type | Y |
| 546 | Patient 25 | NRAS | Q61H | wild type | wild type | Y |
| 547 | Patient 25 | NRAS | Q61K | wild type | wild type | Y |
| 548 | Patient 25 | NRAS | Q61L | wild type | wild type | Y |
| 549 | Patient 25 | NRAS | Q61R | wild type | wild type | Y |
| 550 | Patient 25 | BRAF | V600E | ND | wild type | NA |
